# Optimizing industrial transport with a connected automated vehicle demonstrator for assembly systems and end-of-line production

**DOI:** 10.1038/s41598-024-58627-1

**Published:** 2024-04-05

**Authors:** Luis A. Curiel-Ramirez, Tobias Adlon, Peter Burggräf, Ricardo A. Ramirez-Mendoza, Moritz Beyer, Denny Gert

**Affiliations:** 1 Laboratory for Machine Tools and Production Engineering (WZL), RWTH Aachen University, 52074 Aachen, Germany; 2 Tecnologico de Monterrey, School of Engineering and Sciences, 14380 Mexico City, Mexico

**Keywords:** Autonomous driving, Intelligent transport systems, Smart factory, Vehicle manufacturing, Self-driving vehicle, Connected automated vehicle, Engineering, Electrical and electronic engineering, Mechanical engineering

## Abstract

In recent years, the automotive industry has witnessed significant progress in the development of automated driving technologies. The integration of advanced sensors and systems in vehicles has led to the emergence of various functionalities, such as driving assistance and autonomous driving. Applying these technologies on the assembly line can enhance the efficiency, safety, and speed of transportation, especially at end-of-line production. This work presents a connected automated vehicle (CAV) demonstrator for generating autonomous driving systems and services for the automotive industry. Our prototype electric vehicle is equipped with state-of-the-art sensors and systems for perception, localization, navigation, and control. We tested various algorithms and tools for transforming the vehicle into a self-driving platform, and the prototype was simulated and tested in an industrial environment as proof of concept for integration into assembly systems and end-of-line transport. Our results show the successful integration of self-driving vehicle platforms in the automotive industry, particularly in factory halls. We demonstrate the localization, navigation, and communication capabilities of our prototype in a demo area. This work anticipates a significant increase in efficiency and operating cost reduction in vehicle manufacturing, despite challenges such as current low traveling speeds and high equipment costs. Ongoing research aims to enhance safety for higher vehicle speeds, making it a more viable business case for manufacturers, considering the increasing standardization of automated driving equipment in cars. The main contribution of this paper lies in introducing the general concept architecture of the integration of automated driving functionalities in end-of-line assembly and production systems. Showing a case study of the effective development and implementation of such functionalities with a CAV demonstrator in a more standardized industrial operational design domain.

## Introduction

Transportation systems and human mobility are currently in an age of digitization and flexibilization. Vehicles used for the transport of cargo or humans focus on electromobility elements towards autonomous driving (AD). To achieve this, new urban mobility concepts must be generated to integrate all these digital services into a smart electromobility ecosystem seen from the assembly and production line^1–3^.

The largest automakers have an unusually large global footprint, selling vehicles to consumers and businesses around the world. These large companies are primarily based in a few countries that lead the industry (Japan, Germany, and the United States), but the top 10 list also includes one automaker from Italy and one from South Korea^4^. Nowadays, this market is slowly transforming towards electric vehicles (EVs). In recent studies, global EV sales reached 6.75 million units in 2021, 108% more than in 2020. The global share of EVs (BEVs and PHEVs) in global light vehicle sales was 8.3%, compared to 4.2% in 2020^5^.

Part of this evolution of the automotive industry is accompanied by an increasing amount of automated or assisted driving functionalities. These technologies are also starting to become a big market for the industry. The global autonomous car market size is expected to grow from 20.3 million units in 2021 to 62.4 million units in 2030, at a compound annual growth rate (CAGR) of 13.3%. The growth of this market is driven by the increasing demand for driver assistance systems and stringent safety regulations for a safer driving experience^6^. It is also projected to grow from $1.64 billion in 2021 to $11.03 billion in 2028 at a CAGR of 31.3% in the forecast period^7^. Making it one of the fastest-growing markets in the coming years.

Studies indicates that 9% of manufacturers have adopted autonomous or semi-autonomous mobility, with 10% expecting to follow suit. The key drivers for implementation are cost advantages (86%) and customer expectations (47%). However, 60% cite cost as a major barrier and 42% the immaturity of the technology. PwC mentions the potential 30% savings in total transportation costs for manufacturers by 2040 with aggressive adoption of autonomous driving technologies^8^.

Enabling these new functionalities in nowadays cars opens up an area of opportunity for the integration and use of these technologies within production logistics. The major benefits of these functionalities can be used mainly at the end-of-line production transport. The automation of vehicle movements by self-driving automobiles not only enables a significant reduction in personnel costs in vehicle logistics. The computer-controlled vehicles also promise damage-free re-parking at the highest packing densities^1,2^.

In the context of AD standards, the navigation space inside the factory and at the end-of-line production transport can be conceived as an operational design domain (ODD) for the vehicle. The ODD represents the physical, digital, and atmospheric environments in which autonomous vehicles (AV) need to operate. It’s a way to articulate the space in which the vehicles move, as well as how technology should be built to operate within that space^9^.

This work has two aims, first the conception and automation of the driving of the prototype electric vehicle. Presenting the capabilities and functionalities that it has within controlled industrial environments. Second, to present the industrial ODD and the proof of concept of the integration of the autonomous driving functionalities within the factory. Providing greater flexibility and automation in line-less assembly systems and end-of-line production transport.

The following section presents a review of the state-of-the-art and related work. We expose the necessary and currently used technologies that allow the integration of autonomous driving functionalities within the factory and that were mainly used for the development of this work. In Sect. "Methods" we present the methods and development of the work. We introduce the general logistics of the concept to be tested and its main elements. We describe the architecture for vehicle driving automation and the testing of the localization and control modules. In Sect. "Results" we present the final test scenario and the overall results obtained for the autonomous driving functionalities. We report the results of simulations and driving tests of the car inside and outside the factory. In Sect. "Discussion" we discuss the results obtained and the contribution of this work. Finally, in Sect. "Conclusion and future work" we present the conclusion of the work, validating the proposed objectives; we also introduce some future works that will consolidate the results obtained so far.

## Related work

### Car assembly systems

Over the past few years, the automotive industry has experienced significant changes in the car manufacturing process. The traditional approach of manual labor has shifted towards automation with robots operating on long conveyor belts, which has dictated factory layout for many years. However, the arrival of electric cars and autonomous driving has brought about a shift in the way cars are assembled and the factory structure itself. The traditional fixed, conveyor-driven plant layout is being replaced by a more flexible or line-less assembly layout, as shown in recent studies^1,2,10–13^. Simulation studies have shown promising results for the implementation and evaluation of line-less assembly and hybrid factory layouts, indicating increased workstation utilization and throughput, even considering potential workstation failures^11,12,14^. The proposed uncertain multilevel programming model and intelligent algorithm provide a solution to the vehicle routing problem by minimizing the waiting times of both customers and vehicles under uncertain travel times^15^.

Currently, automakers are producing electric cars with automated driving capabilities that are ready to navigate at the end of the assembly line^16^. This entails assembling all necessary hardware and software components beforehand, and the revolutionary concept of utilizing autonomous vehicle “drive readiness” on the assembly line has the potential to revolutionize the manufacturing process, as autonomous driving in a structured and known environment necessitates fewer regulatory requirements, making it easier and quicker to implement^16^. BMW’s “Autonomous Driving in the Plant” initiative, an innovative project in collaboration with Seoul Robotics and Embotech, exemplifies the automotive industry’s move towards incorporating autonomous systems in manufacturing processes. As a notable success of the BMW Startup Garage, which evaluates potential projects with startups, this effort reflects a broader industry trend towards adopting innovative technologies in production environments^17^.

Implementing line-less assembly, as well as hybrid factory layouts, has the potential to reduce manufacturing operating costs by eliminating the need for a fixed conveyor-type structure and labor to some extent, while reducing space and logistics requirements for manufacturing cars, according to recent studies^1,10,18,19^. This layout introduces greater flexibility into the production process, demonstrating promising results in terms of cost reduction and increased efficiency^1,10^.

Recent studies complementing the general study results include ontology-based modeling and automated deployment of digital twins for manufacturing systems^18^, simulation-based potential analysis of line-less assembly^11,12^, industrial applications of modular software architecture for line-less assembly^12,13^, simulation-based optimization of product integrations^13,19^, and a framework for potential analysis with AutoML^19,20^. These studies, containing various aspects of line-less assembly systems, contribute to the understanding of the benefits and challenges associated with the evolving landscape of automotive manufacturing.

### Self-driving vehicle platforms

The introduction of self-driving vehicle platforms (SDVP) with their own production line leads to significant investments, which drive automakers away due to uncertainty of demand and risks arising from lower technological maturity. The development of new vehicle platforms within assembly lines with a certain level of autonomy opens the opportunity to generate a more realistic concept towards this new panorama^10,21^. Even, the conversion and driving automation of nowadays cars through robotic systems and external actuators allows the generation of new self-driving platforms for testing in defined environments^22,23^.

The development of SDVP is being carried out by numerous companies, universities, and research centers around the world, also generating a close collaboration between them. Some examples of this kind of vehicle platforms used for the research, development, and education of these technologies are BRAiVA, ISEAUTO and UNICARagil^24–27^. These efforts have led to a standardization of manufacturing to some extent. For autonomous driving, a car requires various sensors for the perception of its environment, e.g. cameras, radars, lasers, and ultrasonic sensors. It also required localization and odometry systems such as GNSS, inertial measurement units (IMU), and wheel speed sensors^28–30^.

For the control and actuation of the vehicle dynamics, robotic systems added to the vehicle or already integrated X-by-wire systems are required^31^. This allows us to justify the use of several of these systems, which are currently used in the vehicle (e.Go Life car) in this work.

### Factory navigation technologies

Software plays as crucial a role as hardware to drive these SDVPs as a tool in the factory hall. In aspects of mobile robotics to move the vehicle in the factory, SLAM (simultaneous localization and mapping) algorithm is a basic need. Research has been carried out for years to optimize existing algorithms and for the generation of new algorithms with the help of artificial intelligence (AI). However, due to the complex structure of manufacturing plants, especially because of the repetitiveness of elements inside, there are not many algorithms that work satisfactorily in such indoor environments^32^. Several algorithms have been developed for a specific use case or a specific environment or with a specific type of sensors^33^. This allows us to visualize the complexity of generating an optimal solution for the factory environment.

In recent years, the emergence of automated guided vehicles (AGVs) and autonomous mobile robots (AMRs) in manufacturing plants has led to the development of algorithms specifically tailored for these applications. Typically, these mobile robotic platforms rely on visual sensors for their algorithms such as stereo cameras, monocular cameras, RGB-D cameras, 2D or 3D LiDARs, among others. Some older algorithms used in this AGV rely on visual markers and features in the environment to simplify the SLAM task^34^, but it is relatively difficult to integrate it into a complex industrial environment effectively.

ORB-SLAM2, RTABMAP, SPTAM, and Cartographer are some of the most suitable algorithms for the application among the wide variety of SLAM algorithms available^35,36^. Although these algorithms work well, the final selection depends largely on the factory and the sensors used in the vehicle. Some works^33,37^ have exposed that the algorithms perform differently when evaluated indoors and outdoors. Algorithms such as RTABMAP and Cartographer work with cameras and LiDAR and their performance also varies depending on the input sensor chosen. These algorithms are currently used in the prototype vehicle to partially solve the SLAM task.

Having the best SLAM algorithm available is not enough for vehicle navigation in an indoor environment. Some type of external positioning system is also required to improve localization. A common approach is to have a wireless sensor system. These sensors are useful to SLAM algorithms to improve position estimation^38^. Although these sensors are commercially available, their measurements tend to be noisy. The implementation of some sort of filter becomes a necessity in this scenario in order to reduce noise and fuse various position estimation inputs^39^. Some examples of well-proven and implemented filters are EKF (Extended Kalman Filter), UKF (Unscented Kalman Filter) and Particle Filter^40^.

The current software implementation for the prototype car for this work uses open-source ROS (Robot Operation System). Although the requirements to drive the vehicle in a factory are minimal, some of the requirements are very crucial and important like real-time performance, reliability, adaptability, and interoperability. While ROS lacks the capability in some respects, ROS2 offers better performance regarding these factors^41^. ROS2 also consists of packages with standard algorithms. One of these packages is Navigation2, which contains many of the standards and most widely used algorithms for navigation^42^ has also shown some promising results when deploying the Navigation2 stack with ROS2.

The development of autonomous vehicles has allowed the creation of simulation software frameworks that allow the evaluation of this type of technology in defined environments such as CARLA, PGDrive, SVL, and AirSim. Several of these frameworks even allow the implementation of Hardware in the Loop (HIL), Driver in the loop (DIL), or their variations, enabling an active interaction between real and simulated environments and elements^43^.

### Complementary technologies

To set up a car as a tool on the assembly line, several sensors are required. The earlier sensors are installed on a car, the sooner it can become autonomous. An important component in this step is to have a high-performance computer (HPC) to run algorithms, preferably with GPU(s) to speed up graphics processing. To get rid of this complex control unit and make a car autonomous on the assembly line, cloud-based indoor navigation approaches are also being developed. In this approach, the sensor data is transmitted to the cloud or edge device, all the complex calculations are executed there, and in the end, the control command is sent back to the vehicle^44,45^. The emerging 5G technology will be an important factor to implement this approach with ease since it is a technology that allows various applications in industry 4.0 due to the high capacity, speed, and low latency of data transmission^46^. This approach also has the advantage of easy maintenance of the control logic and updating of the algorithms^47,48^.

To optimize the localization and communication modules in the vehicle, a sensor system based on Ultra-Wide-Band (UWB) technology is used, which has many advantages over other technologies such as Bluetooth or WiFi. Farahsari et al. describe some of its properties, such as accuracy, precision, low power consumption, and low latency^49^. UWB can achieve a positional accuracy of tens of centimeters. It also consumes much less power, which facilitates its use for a long time. In addition, UWB can also be used for geofencing, which can restrict the movement of vehicles in unauthorized or dangerous areas based on the position data received from sensors^50^.

However, in the factory environment, where there is a lot of signal interference and obstacles due to metallic objects, it is advisable to use several transceivers to improve performance. In^51^ some fruitful results are shown with at least four transceivers, which provide a basic setup for the localization of the vehicle within the factory environment^52^ provides a solution using Machine Learning, they tested the system in a factory-like environment and the results showed no appreciable improvement compared to standard Production Data Management(PDM)-based methods.

## Methods

### Autonomous vehicle transport concept

**Figure 1:**
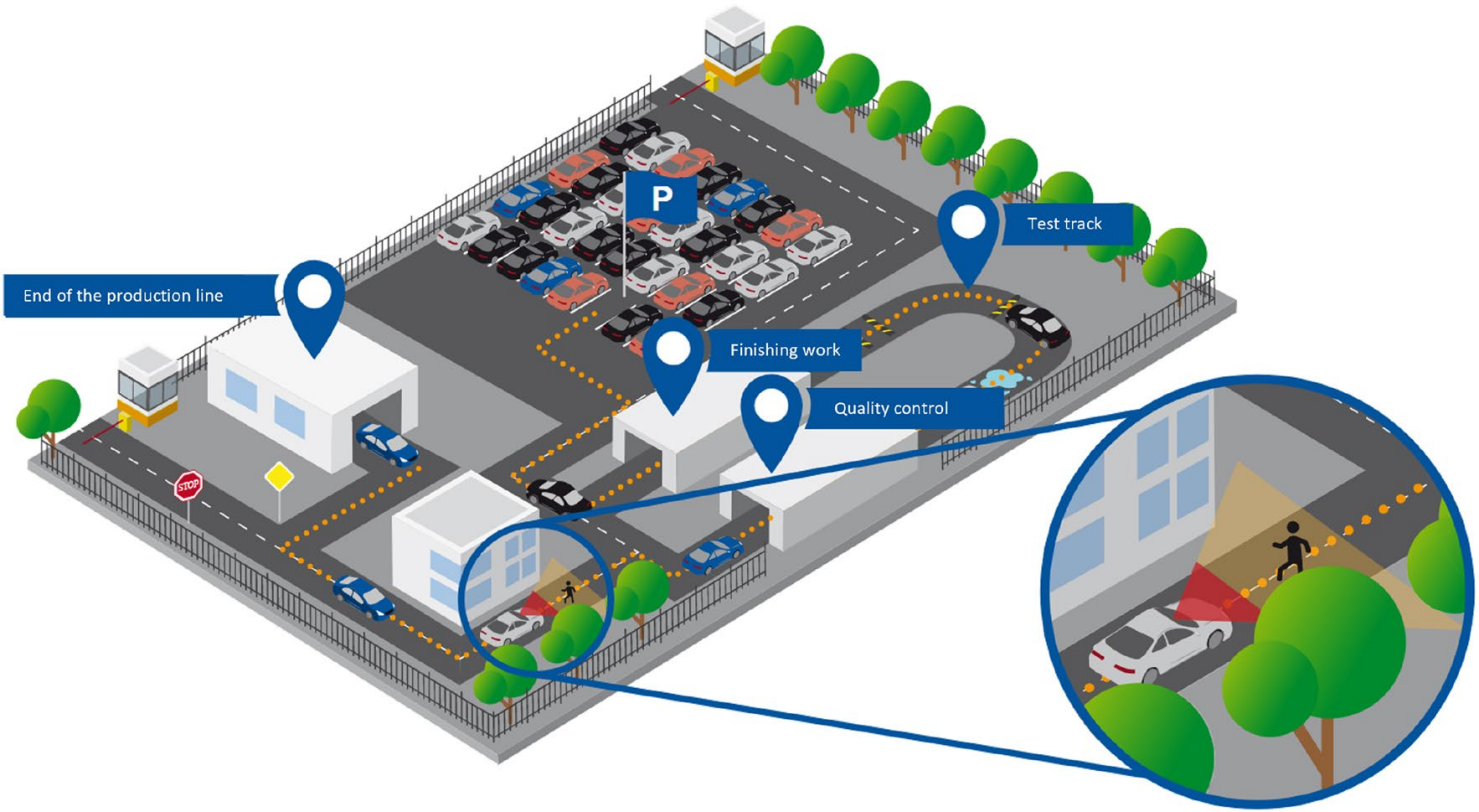
Automated vehicle transport study case. The vehicles run automatically from the end-of-line, and can automatically carry out a test drive to the parking lot or for loading on the ship, train, or truck (Figure provided by WZL-RWTH Aachen University).

The goal of the work is to make the material flow more flexible during assembly and subsequent distribution within the factory facilities. For this, driverless transport systems with a high degree of autonomy are used. For late assembly steps and after the end of the belt, the vehicles are enabled to navigate driverless. This will be accomplished using standard low-speed automation equipment. This seeks to validate the study case of automated vehicle transport in industrial environments (see Fig. 1).

The central component of this work is the driving automation concept of an electric vehicle that does not have any type of preinstalled sensor to provide AD functionalities in it. The benefits in terms of economic and safety elements of this concept on the production line were investigated^1^. Complementary functionalities of the vehicle were also developed and tested, such as collision detection in industrial environments (real and simulated)^53,54^, the communication infrastructure and the generation of control commands from the Control Station (CS) are defined and tested. Simultaneous localization and mapping (SLAM) functionalities are developed within the factory and the motion control of the vehicle utilizing an X-by-wire interface is validated.

### Functional architecture

The results of the development of vehicle-integrated systems that enable self-driving functionalities and systems that complement functionalities for the overall work were gathered. The prototype vehicle was built on an e.GO Life as a self-driving vehicle platform. In the first phase of the work, a new assembly sequence was defined based on the requirements of the self-driving vehicle platform, which maximizes the proportion of automated stations in the total added value of the assembly. All drive and control components had to be put into operation as quickly as possible for the development of driving tests.

**Figure 2:**
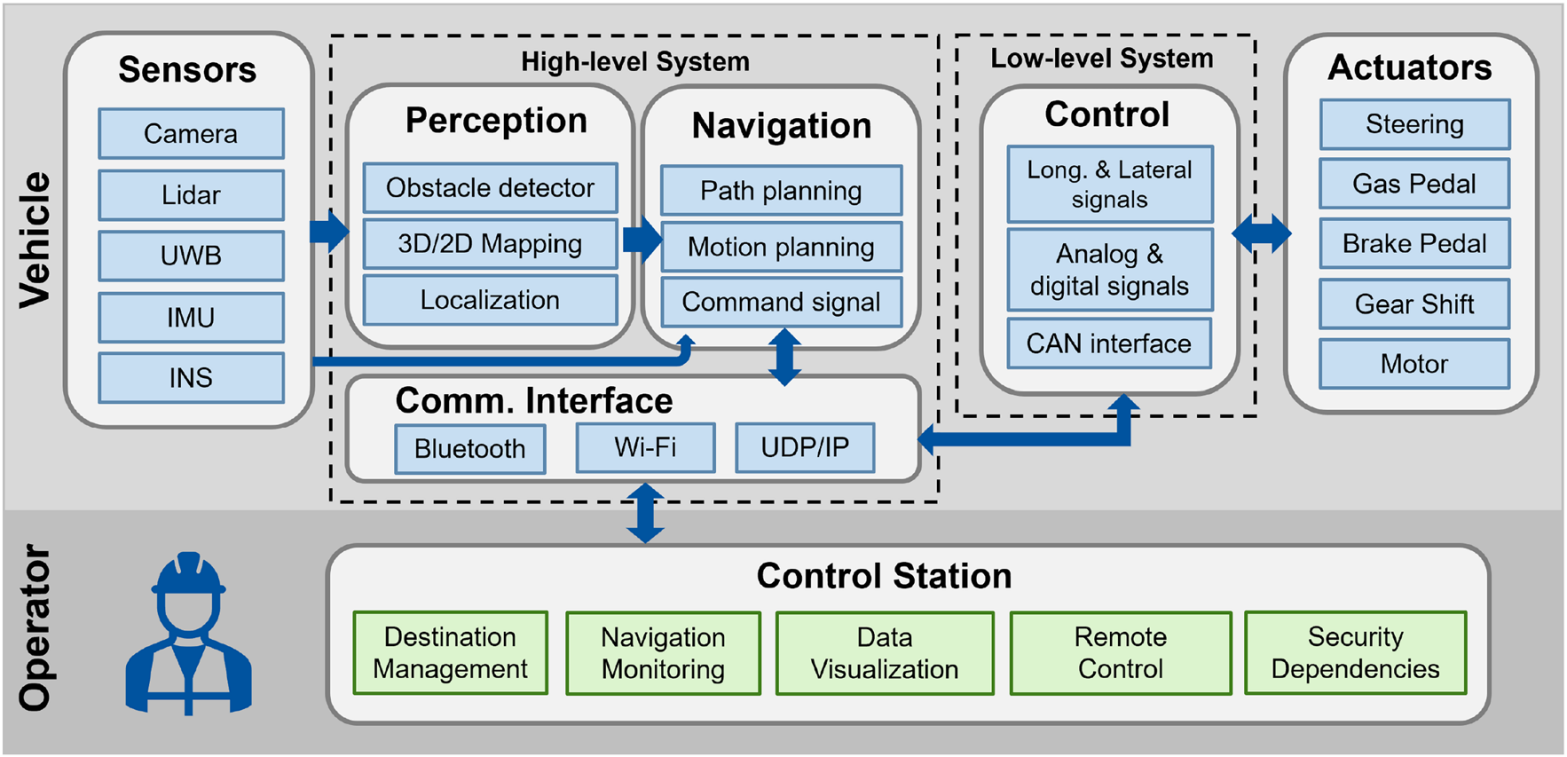
General functional architecture for the CAV demonstrator.

The technical implementation of the automated driving of the SDVP was carried out by different control modules presented in a functional architecture (see Fig. 2).

These modules integrate the temporarily installed components necessary for the vehicle’s automation and data processing through various sensors and high and low-level systems. The high-level system (HLS) uses a simultaneous localization and mapping (SLAM) algorithm to calculate the current position of the vehicle based on the perceived environment. It also handles communication with the control station (CS) for complementary functionalities. Finally, the low-level system (LLS) is connected to the actuators allowing the motion control of the vehicle in the final demonstrator. The driving automation architecture designed for the vehicle allows the perception and motion control functionalities. The car has mounted lidars, a camera, inertial measurement units (IMU), an Ultra Wide Band (UWB) receptor, and a high-precision Inertial Navigation System (INS) for processing the perception of the environment, localization, mapping, and odometry data. The control of the car is achieved through two modules, the HLS performs the task of localization, mapping, and motion planning. This module sends direction and speed setpoints to the LLS, which then utilizes closed-loop control to stabilize the vehicle (laterally and longitudinally) according to these setpoints. The LLS allows the manipulation of the steering wheel, pedals, and gear of the prototype car for driving automation.

The results obtained by these elements allow us to demonstrate the concept of driverless transport systems at the end of the assembly line. However, the concept must be adapted to the needs and capabilities of the production and logistics of the car in the factory, which could make the concept very difficult to manage and control.

### Connected automated vehicle demonstrator

For this work, an overall concept for the automation of vehicle logistics is developed. For the safe automation of the vehicles on the factory premises, the functions of collision protection, localization, communication, and actuators are dealt with in detail. A novel control system processes safety-critical information.

Between low-cost (e.g. e.GO Life) and high-cost (e.g. Porsche Macan) vehicles. High-cost vehicles have a large number of internal sensors that can be used for collision protection and localization. For poorly equipped low-cost vehicles, temporary, external sensors may be analyzed and tested.

#### Hardware setup

For the technical implementation of such a cyber-physical system, such as self-driving vehicles, sensors are needed to perceive the environment. Safe control of the vehicle becomes possible if both, the vehicle and obstacles, can be reliably located. Based on the environmental information, the movement can then be planned and communicated to the vehicle’s actuators, which finally implement it. The functions of environmental perception and motion planning can either be fulfilled by the vehicle’s own components or provided by the surrounding infrastructure.

**Figure 3:**
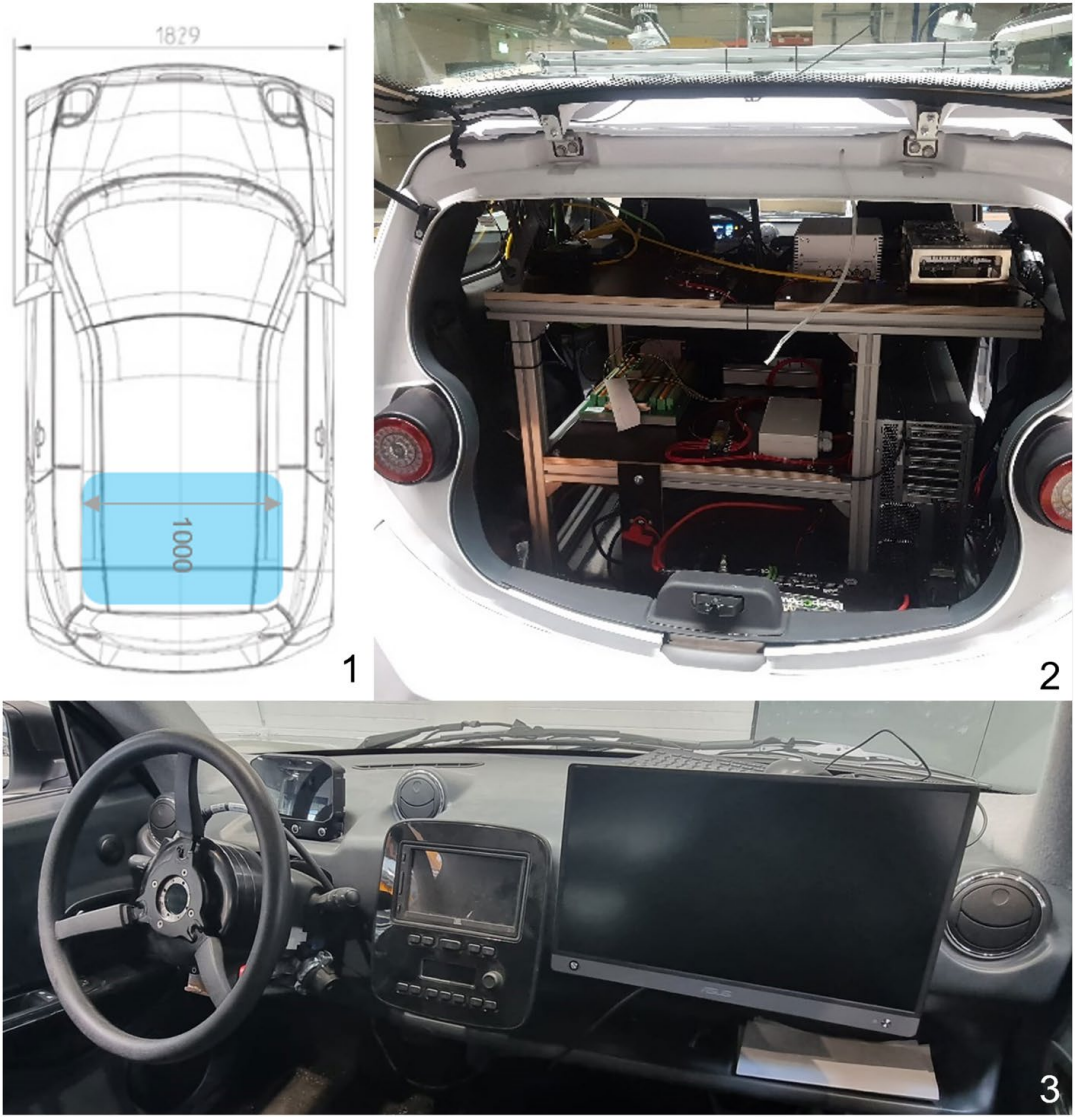
(**1**) Positioning of computer systems inside the vehicle, (**2**) Rack with the hardware setup, (**3**) Steering wheel actuator and screen of the prototype vehicle.

The basis for the processing of work is the automation of a test vehicle. For this purpose, a vehicle from the company e.GO Mobile is available. To prepare, the rear seat of the vehicle is first built out and a rack for corresponding computer systems is provided (see Fig. 3). Furthermore, the electrical-electronic equipment of the vehicle is designed, and its implementation plan.

For the actual automation, a DSpace Micro-Autobox II is installed as a low-level system (LLS). Furthermore, a device for mechatronic control of vehicle steering is implemented. A technical interface for onboard control of ignition, brakes, and gas is also developed and installed. A high-performance computer (HPC) is installed as a high-level system (HLS) to calculate paths and safety-relevant decisions. Sensors are also used to measure lateral and longitudinal acceleration.

#### Sensor setup

**Figure 4:**
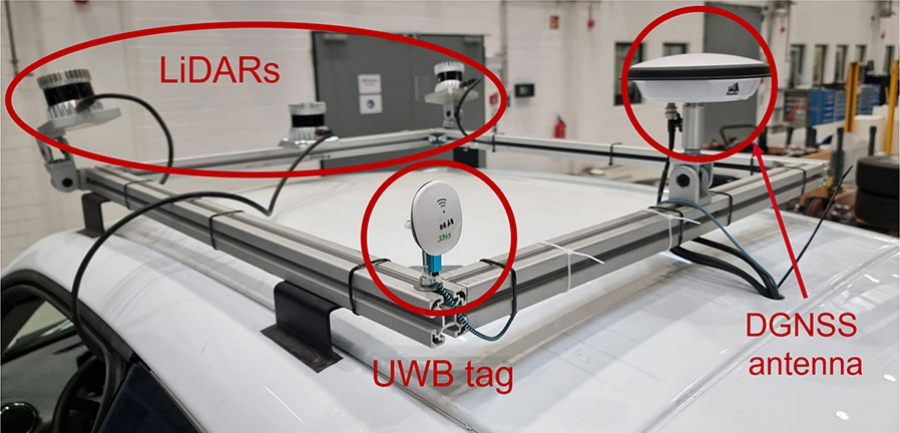
Setup of temporary and external sensors for the prototype vehicle.

In the automated transport of manufactured vehicles, they must be driven independently to the factory parking lot after the end of the assembly line. To automate the driving task, the central functions of locating vehicles inside the factory and outside to complete the task must be fulfilled. To ensure cost-effectiveness, the functions must be implemented without additional sensors or hardware for data processing. For high-cost vehicles, the localization must be carried out with the sensors already normally integrated (ultrasonic, radar, and cameras). For testing purposes, the prototype vehicle is carried out through a setup of temporary and external perception sensors; in this study case, mainly through LiDARs. Additionally, the localization inside the factory is optimized by fusing the information with UWB modules and outside the factory hall with an RTK-DGNSS module (see Fig. 4).

#### Vehicle localization & mapping

To achieve accurate localization and mapping in our SDVP, we employ a systematic approach mainly using various modules that have been currently developed in ROS and other open-source sources. The localization task is centered on estimating the vehicle position, denoted as vP = (x, y, z), within a global coordinate system of a known environment at any given time tP. These coordinates, x, y, and z, define the 3D positioning of the vehicle. While a map of the area is beneficial, it is not mandatory for localization. The problem is categorized into three areas: local self-location, determining the vehicle’s position concerning a nearby reference point; global self-location, estimating the position relative to a global reference point; and the kidnapped robot problem, addressing re-localization after an unknown displacement.

In local self-localization, where the approximate position is known, recalculating occurs upon vehicle movement. This involves assessing incoming sensor data, predominantly utilizing odometry data and minimizing total error by integrating with other sensors like an IMU. The resulting position is determined within a vehicle coordinate system.

Contrastingly, global self-localization deals with an initially unknown vehicle position, aiming to determine it on a planning map using data from vehicle movements and sensor input. Position determination occurs relative to a world coordinate system, presenting a more intricate challenge than local self-location.

To address these challenges, we developed modules employing mainly lidar sensors on the vehicle to tackle simultaneous localization and mapping (SLAM) problems. Initial testing of these modules was conducted in simulation environments using the open-source SVL simulator. This emulator faithfully replicates the sensor setup currently mounted on the vehicle, providing a robust validation platform for our localization and mapping solutions.

**Figure 5:**
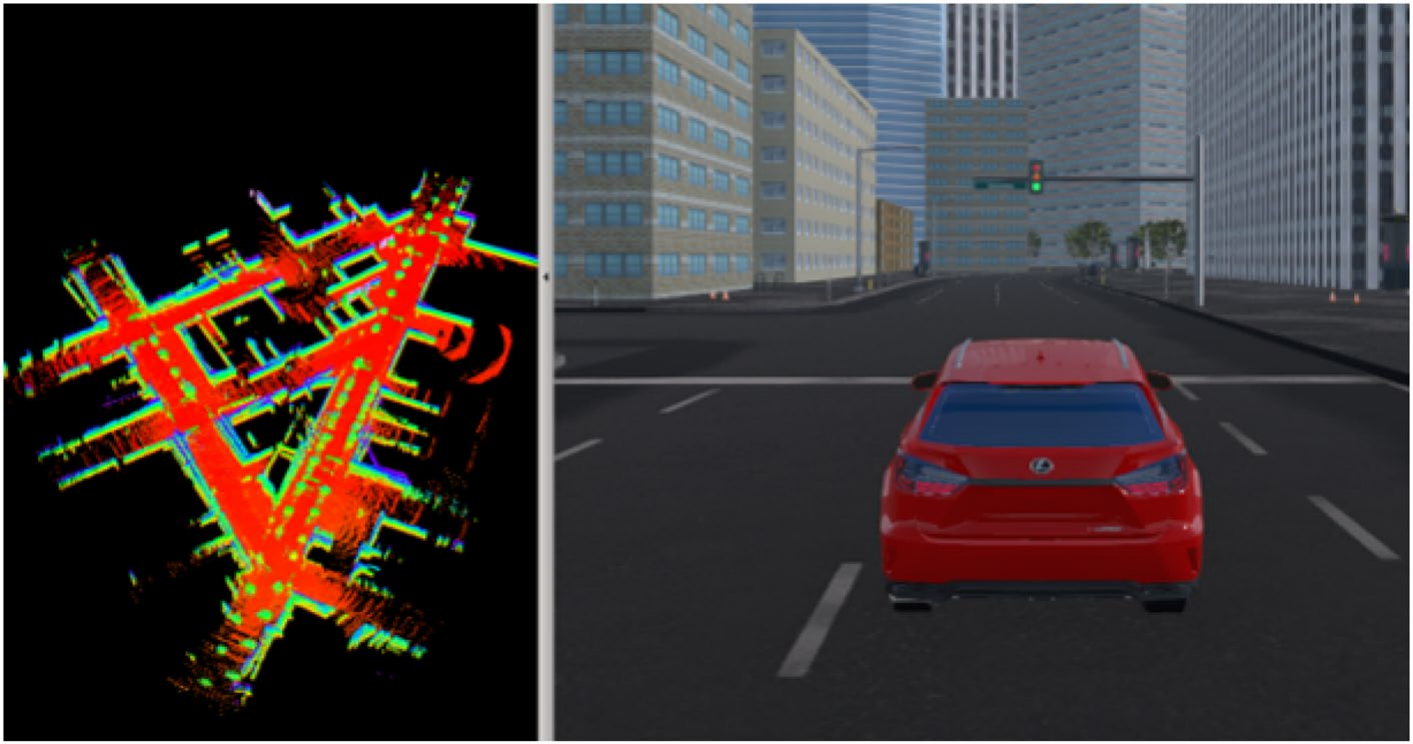
Mapping tests in SVL simulator, example using LeGo-LOAM.

To generate the SLAM, the task was divided into 2 parts, first the mapping and then the localization. Mapping of the environment was generated in order to obtain a 3D and 2D map. Various open-source packages, including Cartographer, Rtabmap, LeGo-LOAM, and Gmapping, were tested for this purpose (see Fig. 5).

**Figure 6:**
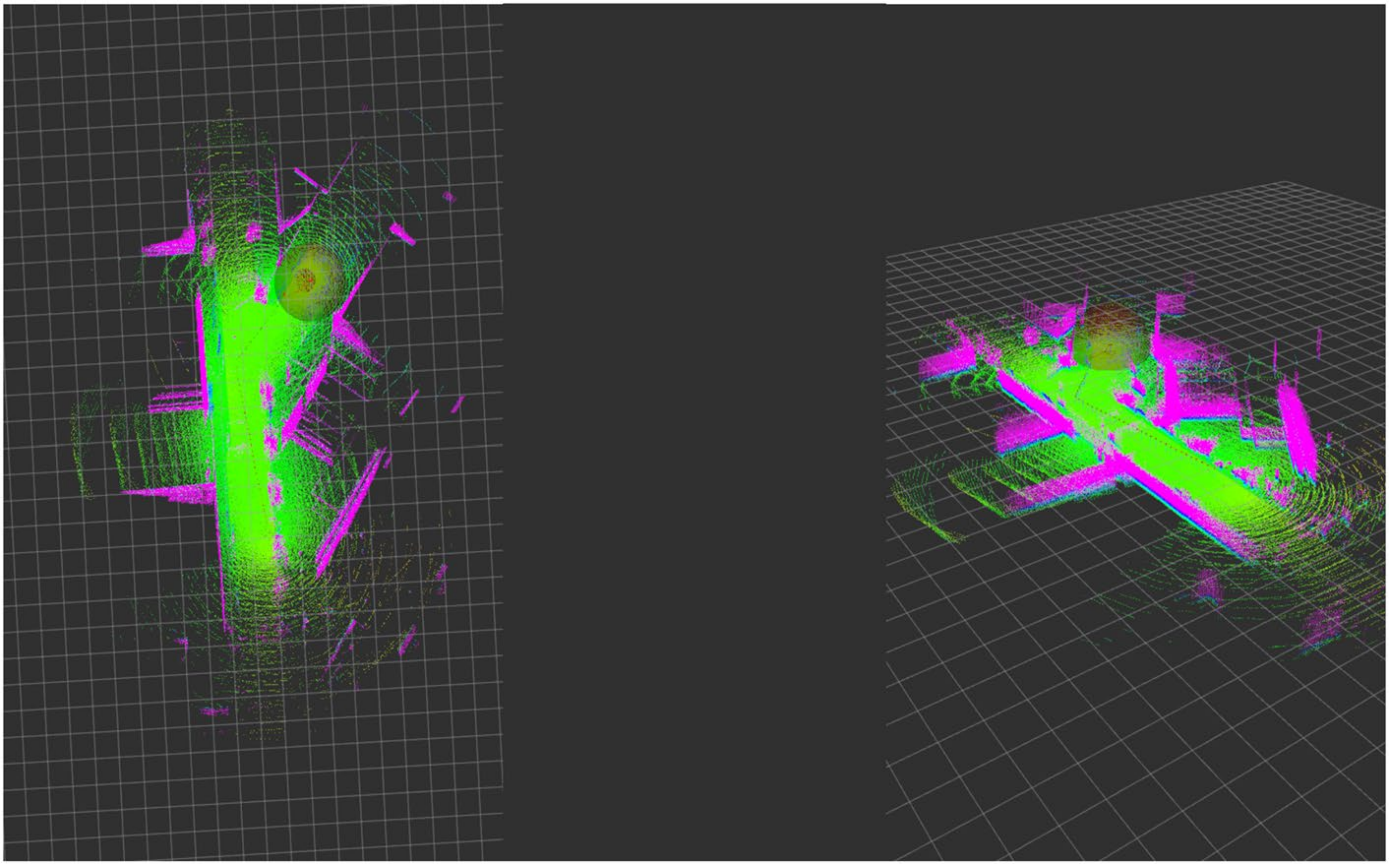
Localization of the vehicle in the SVL simulator using the previously generated map.

Second, the localization of the vehicle on the generated map (see Fig. 6). This requires the fusion of the different emulated sensor data, which in this case was achieved by using multi-input state estimation filters. Extensive testing involved different filters such as extended Kalman filters (EKF), unscented Kalman filters (UKF), and particle filters to address this challenge.

In the mapping phase, Cartographer generated good quality 2D map, but the tuning was complicated for a better result in the 3D map. Rtabmap had the disadvantage of only supporting one lidar source compared to Cartographer, so it was necessary to generate a cloud point merge node. After this, a 2D and 3D map of good precision was generated. LeGo-LOAM gave us a balanced result for the generation of a 3D map. Gmapping provided fundamental tooling for producing a 2D map with high precision.

For the localization phase, we found that Monte Carlo localization (MCL) models, implemented through particle filters, demonstrated superior performance and precision in locating the vehicle. This integration was accomplished using the ROS Adaptive MCL node, which effectively fused information from 3D lidars and other complementary sensors mentioned earlier. Notably, the vehicle’s localization in the simulation environment proved successful on generated maps, with an average error of merely 45 cm in the simulation scale.

**Figure 7:**
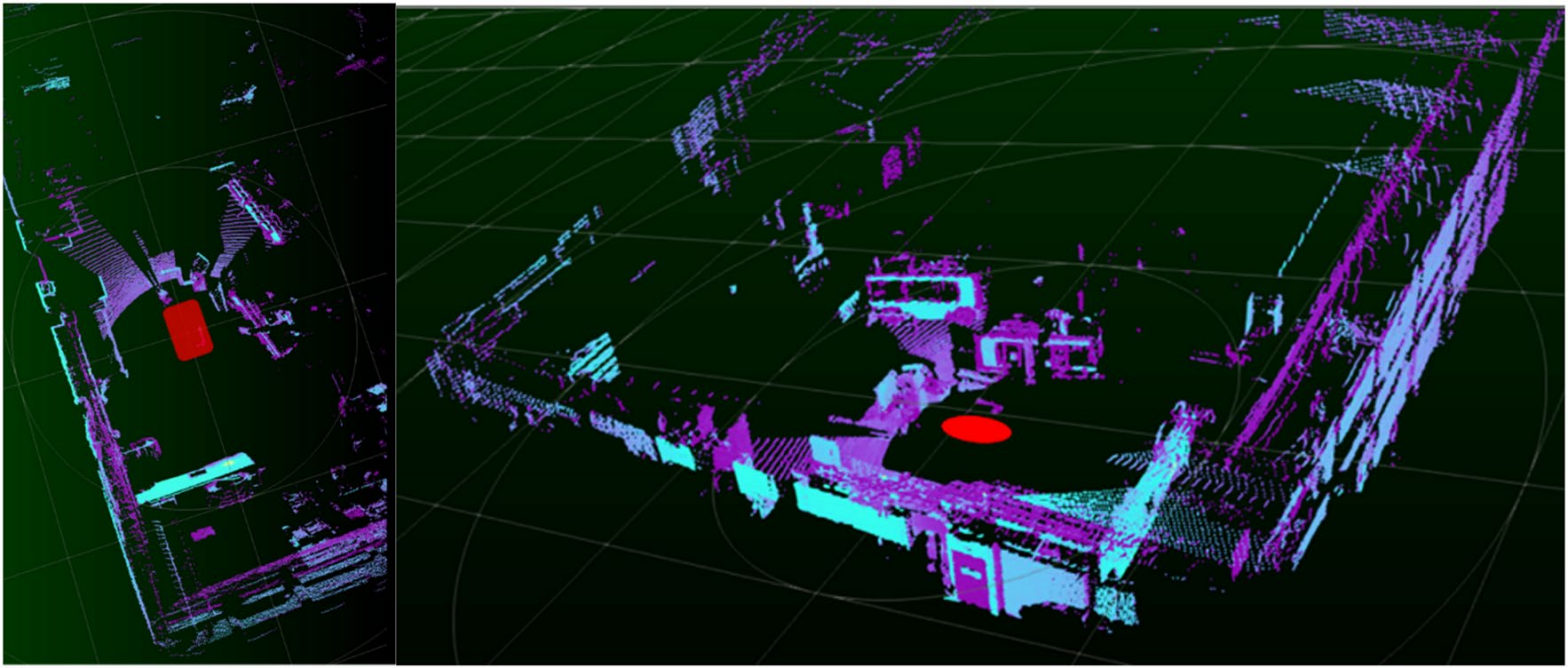
3D mapping tests with the lidar sensors installed in the prototype vehicle.

After validating the SLAM algorithms on simulated environments, the necessary tests continued on the prototype vehicle. The mapping test was first performed using the car’s lidar sensors within the factory environment (see Fig. 7).

**Figure 8:**
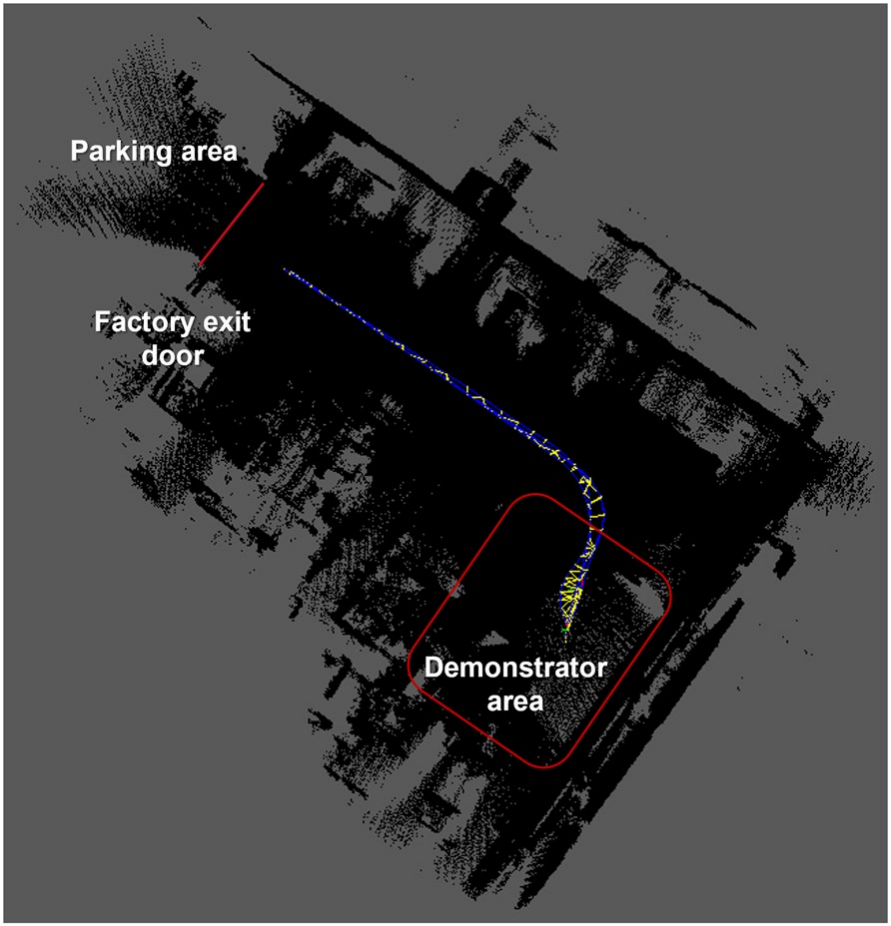
Vehicle localization tests within the factory environment though lidar sensors.

After obtaining a map with enough quality, localization tests were carried out using only the lidar sensors and the state estimation algorithms tested in previous simulations. For the tests, the SDVP is driven on the route proposed for the final demonstrator. The driving and localization of the vehicle are carried out several times with the generated map looking for possible errors in aspects of safety, odometry, communication, or processing (see Fig. 8).

Incorporating odometry as a fundamental navigation method for ground vehicles is crucial in our approach. This technique derives the vehicle’s position from its own motion data, effectively charting the traveled route. Speed and orientation are determined through encoder data or control commands, providing valuable insights into the vehicle’s dynamics. However, it’s essential to note that odometry is not without its challenges. The method can only measure actions, introducing various sources of error. These errors accumulate over longer distances, limiting the suitability of odometry for extended navigation. Mitigating strategies include reducing speed and acceleration to minimize errors and enhance the reliability of this method.

In our prototype vehicle, odometry is generated through the integration of lidar sensors and complementary motion sensors. The lidar sensors precisely capture the vehicle’s movement by measuring the time-of-flight of laser pulses, offering accurate distance measurements. Complementary motion sensors provide crucial data on the car’s acceleration and angular velocity. By doing the sensor fusion of these data, our system calculates the vehicle’s displacement, speed, and orientation over time, constituting the odometry data.

The complementary motion and localization data is obtained and fused in two phases:

1. In the first, the speed and orientation of the car obtained by the car’s CAN bus and the inertial sensors installed in the vehicle are fused. For the localization inside of the factory, this odometry phase gives good but not sufficient solutions for precise and reliable localization due to the cumulative errors in the integration and the limitation to local localization being able to move along the route.
2. In the second phase, it uses the localization data from the UWB modules inside the factory and the RTK-GNSS for the outside as an additional localization source. This information is fused with phase one data to optimize the tracking and localization of the vehicle during its driving.

UWB is a radio-based communication technology for short-range use and fast, stable data transmission. Due to its accuracy, transmission speed, and reliability, UWB is a widely used technology for the indoor localization of moving assets in complex and space-sensitive environments. UWB uses high pulse repetition rates to increase location accuracy and data rates by transmitting more pulses per second. UWB can determine the relative position of other devices in the line of sight even up to 200 meters based on the IEEE 802.15.4a standard.

In the eGo Vehicle, the UWB technology is used in two aspects, the communication of devices through MQTT, as exposed in the Control Station and Communication section (see Fig. 13) and for the localization and tracking of the vehicle within the demonstrator area.

**Figure 9:**
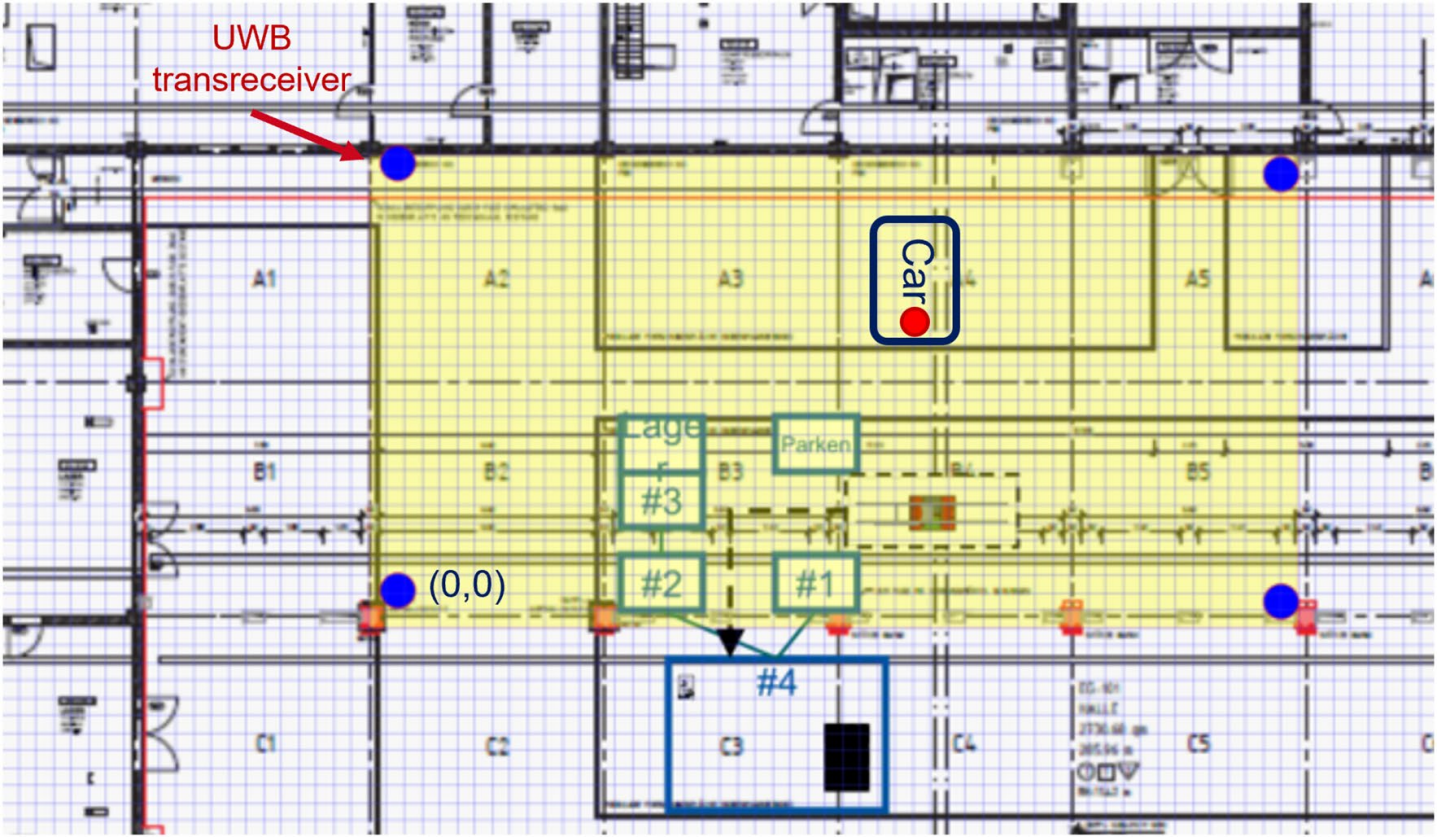
Setup of UWB transceivers inside the demonstrator area.

To archive this, 4 transceiver satellites were installed inside the factory (see Fig. 9). These are communicated to UWB tracking tags (see Fig. 4) to obtain local coordinates within the demonstrator area. The localization data is used to track the trajectory of the car within the demonstrator area and thus subsequently is being fused with the odometry data obtained in phase 1 and improve the localization of the vehicle.

In the general concept of this section, the vehicle must be able to be localized effectively inside and outside the factory. For external localization, it is supported by a Differential Global Navigation Satellite System (DGNSS) with Real Time Kinematic Positioning (RTK) system installed in the prototype vehicle. For the proof of concept in the demonstrator presented in the Autonomous Vehicle Transport Concept section, the driving of the vehicle outside the factory is presented in a complementary work^55^. Therefore, these tests with the RTK-DGNSS systems were not necessary. Even so, the capabilities of the vehicle to be localized outside the factory are exposed, as is presented in the general concept of this section.

The final odometry is obtained through the fusion of data from various sensors integrated into our prototype vehicle. Lidar sensors contribute accurate distance measurements, capturing the vehicle’s spatial displacement. Complementary motion sensors, including accelerometers, gyroscopes, and internal vehicle sensors, provide critical data on acceleration and angular velocity. The integrated system processes this diverse dataset, creating a comprehensive overview of the vehicle’s movement. Employing advanced algorithms and sensor fusion techniques, we synthesize the lidar and motion sensor inputs to generate a robust and precise odometry. This fused odometry information serves as a foundational element for the vehicle’s navigation and control within the industrial setting, ensuring accuracy and reliability in its movements.

#### Control & actuators

**Figure 10:**
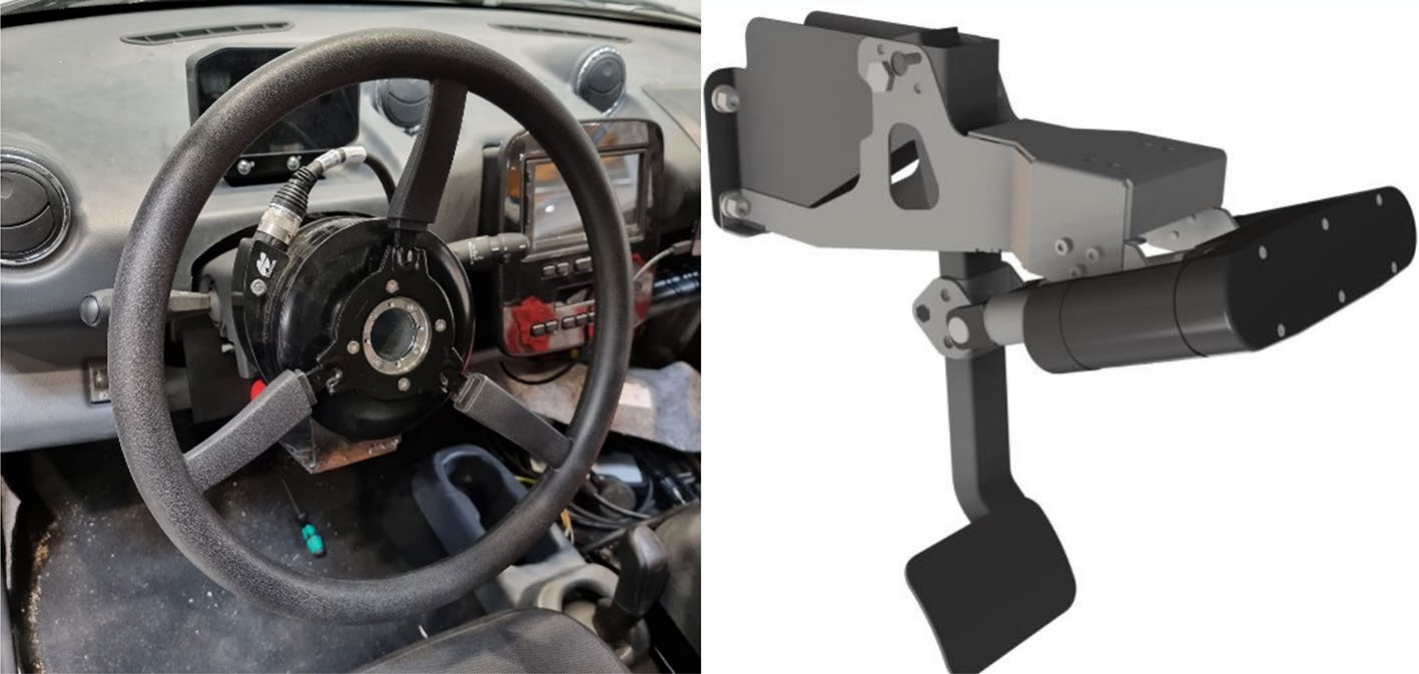
Selected steering wheel actuators and pedals for dynamic vehicle control.

The necessary actuators were selected to automate the steering wheel and pedals (acceleration and braking) of the prototype vehicle, forming a critical component in achieving autonomous driving capabilities (see Fig. 10). The actuator for the steering wheel ensures precise control over the vehicle’s direction, facilitating accurate navigation within the designated paths. Additionally, the actuator for the brake pedal enhances safety and control, allowing for prompt and controlled deceleration when needed. Together, these actuators integrate seamlessly with the vehicle’s control system, enabling comprehensive automation of essential driving functions.

**Figure 11:**
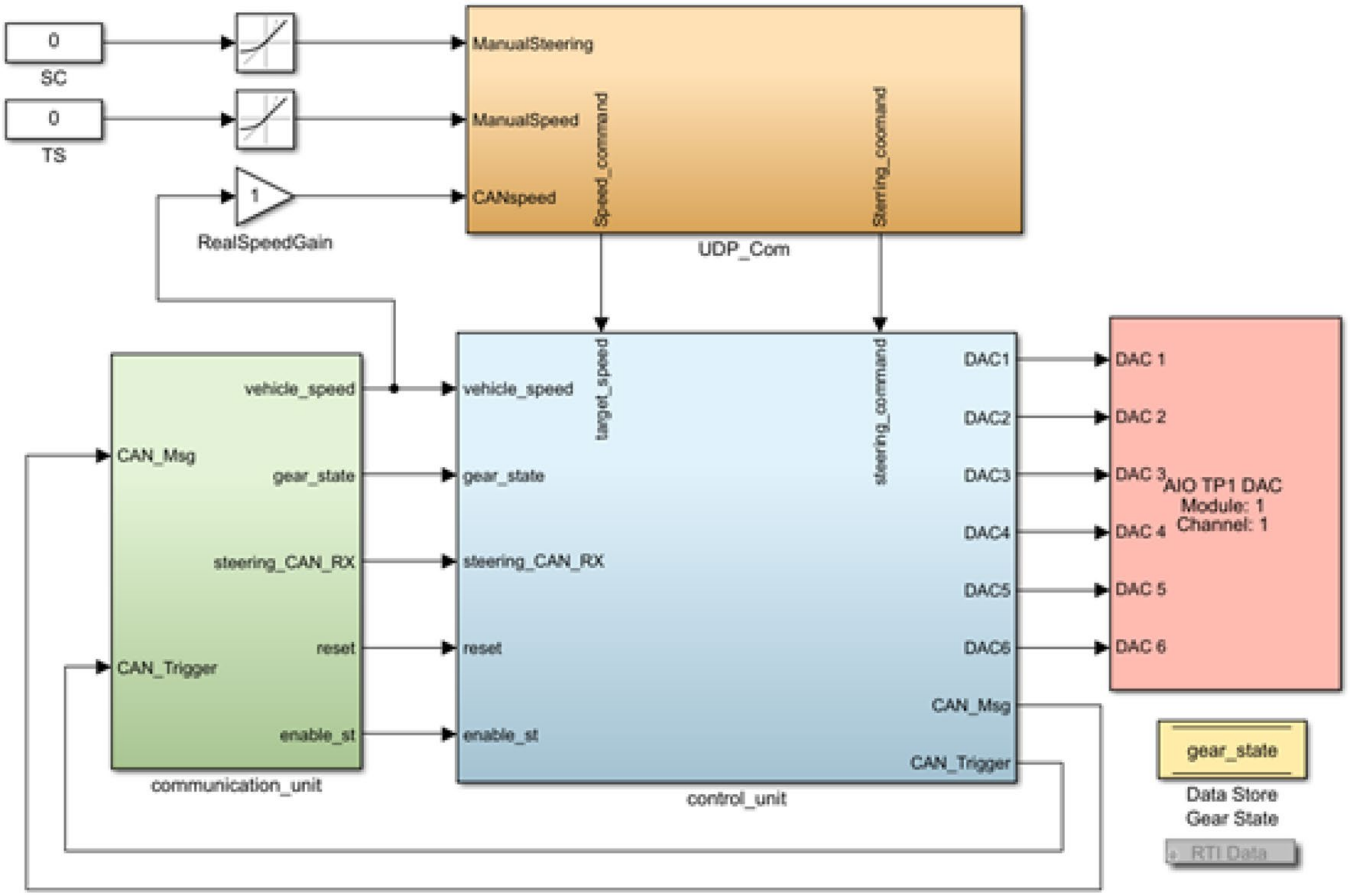
General model of the system for the control of the prototype vehicle actuators.

The control system stabilizes the longitudinal and lateral movement during driverless operation, ensuring a smooth and controlled trajectory. For this purpose, the components of the actuators are controlled with robust control strategies, such as Proportional-Integral-Derivative (PID) algorithms, and Model Predictive Control (MPC). The complexity of the algorithms used was selected based on the performance of the Vehicle Control Unit, in this case, a DSpace MicroAutoBox II. For the design of the system control, dynamic and state variables are considered. These are obtained through the inner CAN bus of the vehicle, for example, the current speed, transmission states, and steering wheel angle, among others (see Fig. 11).

**Figure 12:**
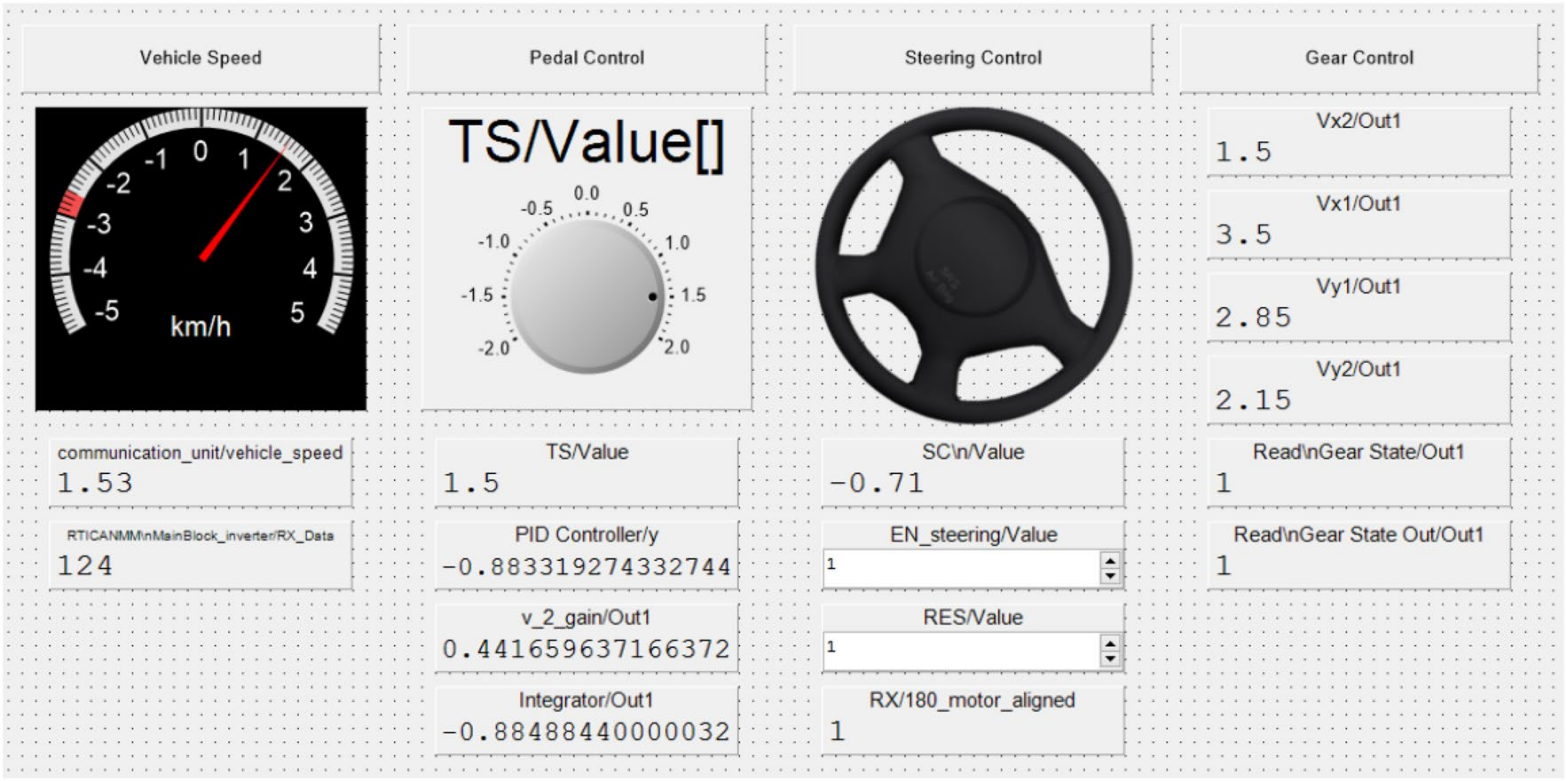
User interface generated to send control commands to the actuators.

These data are connected to the Control Desk interface that allows control of the actuators’ movement. Typically, low-cost vehicles lack a native interface for sending control commands to actuators. To address this limitation, we developed a custom interface, as presented in Fig. 12, to facilitate the generation of essential motion controls for the vehicle.

### Control station & communication

The communication infrastructure between the CS and the autonomous vehicles was developed. Wireless communication systems were added to the overall automation architecture of the car. These are connected to the HLS and enable vehicle-to-infrastructure (V2I) communication within the factory, or more specifically with the CS.

**Figure 13:**
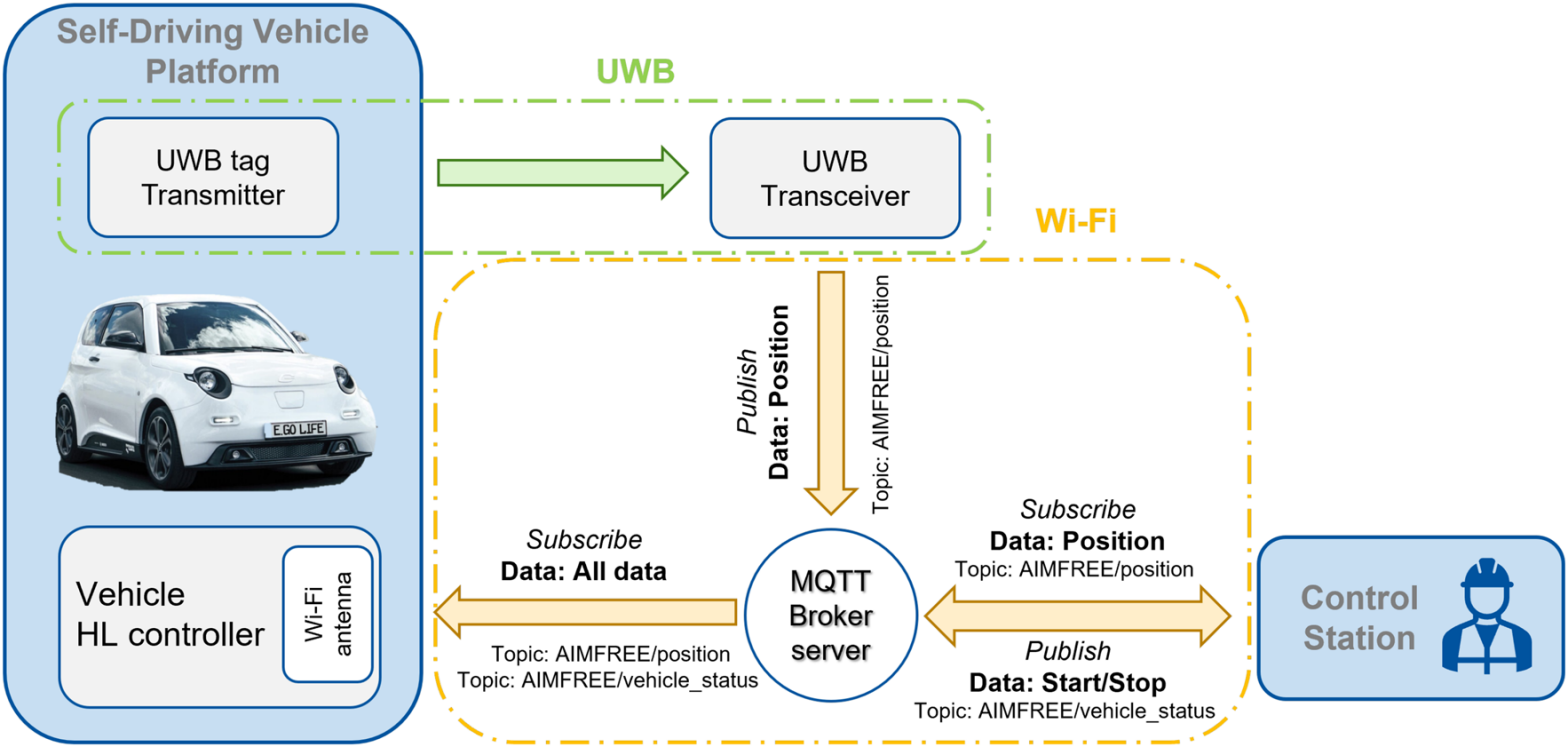
General overview of the vehicle communication using MQTT protocol.

For this, UWB wireless technology provided by Pilz GmbH & Co was used, which allows localization and communication functionalities between nodes. Through Wi-Fi (IEEE802.11n), data transfer and communication between the CS and the test vehicle are achieved. The MQTT protocol is used for communication and generation of control commands between the nodes of the network (see Fig. 13).

The positioning sensor tag is mounted on the vehicle and publishes the data to the MQTT Broker server. MQTT is a lightweight publish/subscribe-based messaging protocol. It is designed to work with the UDP/IP protocol. Each node (MQTT clients) publishes and/or subscribes to the MQTT broker/server on a unit topic. The nodes communicate with each other through broadcast messages on the topic. MQTT is used to communicate the UWB positioning sensor transmitters. The UWB-Transceiver broadcasts the data to the MQTT Broker server on the general topic “AIMFREE/collision-detection/clearances”. The vehicle HLS subscribes to the same topic and receives the data each time the UWB tag transmits a message.

The overall vehicle communication provides information and data transmission support to the collision avoidance, localization, and route planning functionalities for the overall architecture and can be accessed through the CS. During development, tests are performed continuously to ensure the correct behavior of the software and to detect errors in the early stages of development.

#### Emergency stop systems

**Figure 14:**
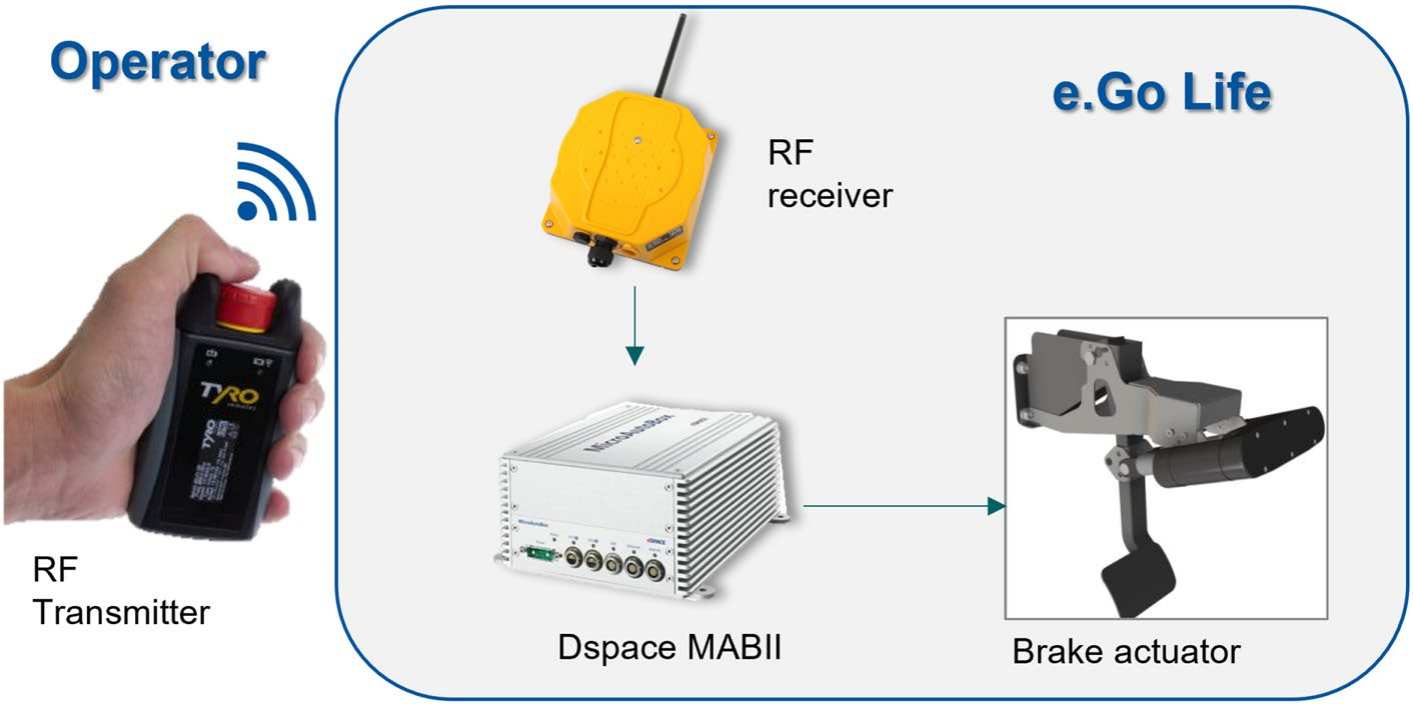
Remote emergency stop system installed in the vehicle.

To cover the security requirements, additional security features were generated, such as the installation of emergency stop buttons inside and outside the vehicle. The automatic driving of the vehicle is carried out at walking speed. This speed is regulated from the factory setting and additionally limited in the control model. Each automated trip is accompanied by a “safety commissioner” (external to the vehicle), who can activate an emergency stop through the remote emergency stop (see Fig. 14).

Dspace MicroAutobox receives the emergency stop signal and sends it to the brake actuator, which is activated to stop the vehicle automatically. This allows the vehicle to be stopped in the event of an error, loss of control, or possible collision situation.

## Results

**Figure 15:**
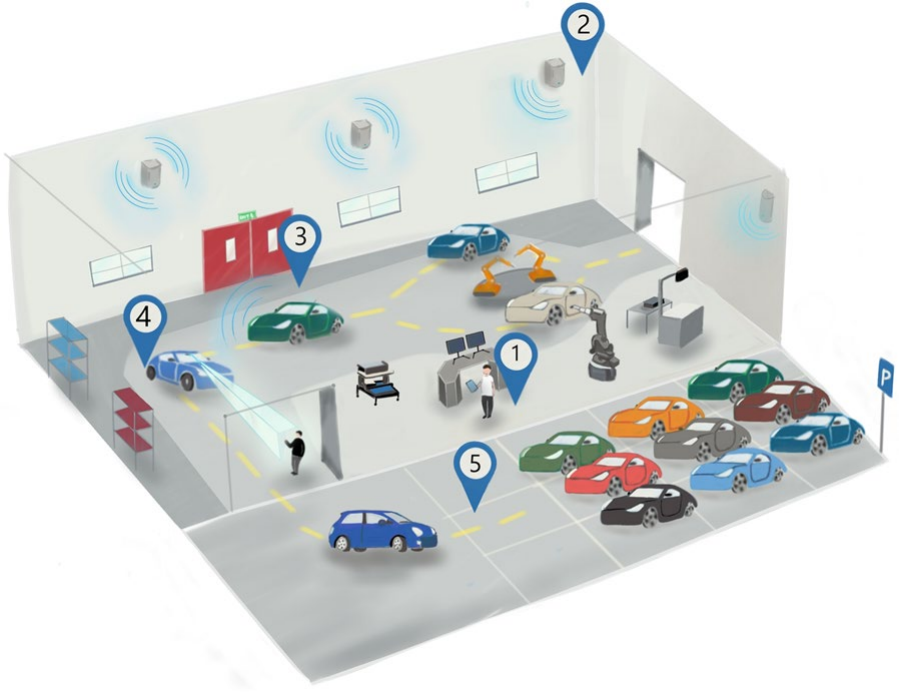
Transport automation components in the factory that are tested during the Demonstrator.

Considering the elements established in the preview sections and the results obtained, the integration of the technologies of the demonstrator in the SDVP was carried out. For this, different components were defined that are tested in the demonstrator. These components are tested within the WZL Machine Hall of the RWTH Aachen University. The tested components are test cases of the general concept presented at the beginning of the "Methods" section in relation to the automation of transport inside and outside the factory. In this case, in the Demonstrator, the prototype vehicle will be tested inside the factory and thereby generate an ODD in which the vehicle can safely navigate. The components that were tested on the Demonstrator are detailed and listed below (see Fig. 15).

1. Any assembly operation is performed on the SDVP (the vehicle must be turned on). Once the assembly operation is completed, the SDVP receives a driving command through the CS and begins driving.
2. The UWB modules enable accurate vehicle localization support within the factory.
3. The SDVP is constantly communicating using the MQTT protocol through the Wi-Fi network within the factory hall to control the driving status.
4. The SDVP travels from the starting position (3) to the parking position (in this study case, the end of the aisle). During the journey, the vehicle integrates the frontal collision detection system through its perception sensors. The vehicle stops if it detects an object in front of it. The vehicle sends an alert signal to the control station via MQTT.
5. If no obstacle is detected, the vehicle continues to the end of the factory hall and stops at the desired location (in the ideal case of transport automation, this would be to the parking lot).

### Driving simulations

**Figure 16:**
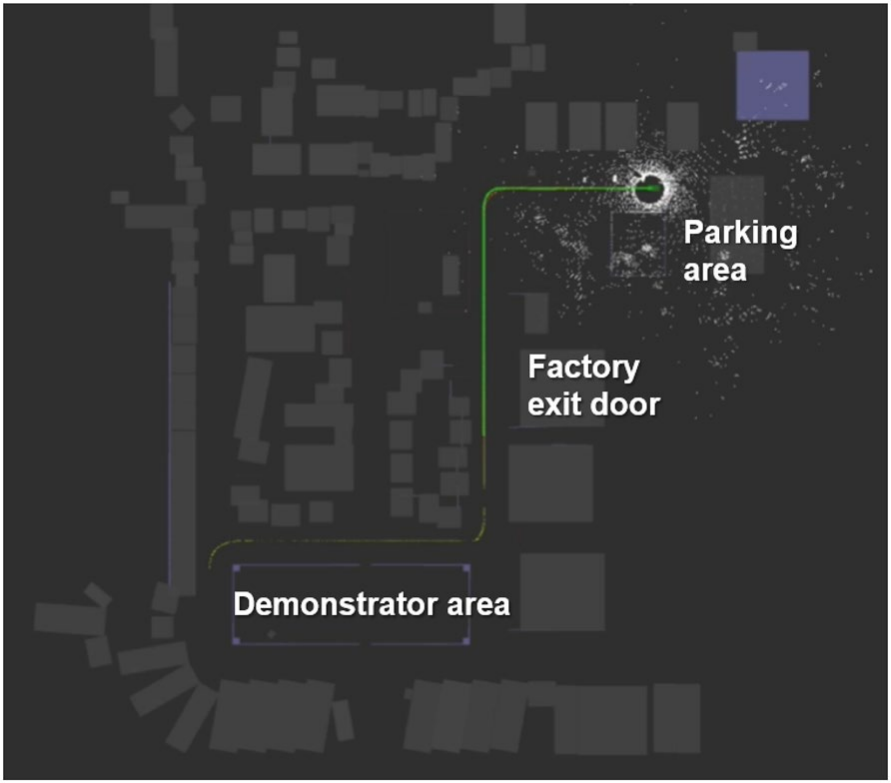
Virtual map in CARLA simulator for vehicle navigation.

The implementation and testing of the complete autonomous driving (AD) stack inside the factory was carried out first in simulation and finally inside the WZL Machine Hall on the established route. For the simulation, the CARLA simulator was used to test the vehicle’s navigation modules. The driving points where the vehicle begins and ends its journey within the virtual environment were defined (see Fig. 16).

**Figure 17:**
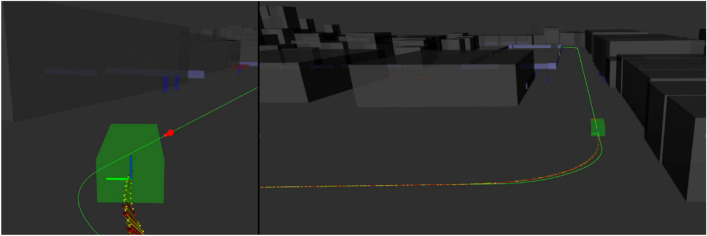
Path planning and motion control tests of the vehicle in the simulated environment.

The system generates the route to follow between the established points. The user sends the start signal and the vehicle begins its journey. The navigation of the vehicle considers a route with straight lines and curves. This validates the motion control of the vehicle to follow the established route (see Fig. 17).

### Final driving tests

**Figure 18:**
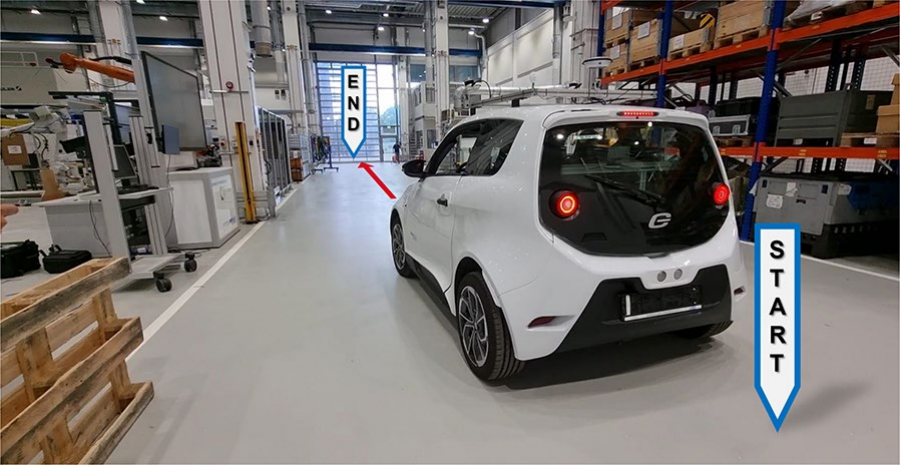
Test drive route of the SDVP inside the factory hall.

After validating the general modules of the AD stack in the simulation environment, the driving tests of the vehicle inside the factory hall began. The driving tests were carried out with the objective of testing and validating the components established in Fig. 15. The safety parameters within the factory hall and the vehicle are reviewed to ensure its total integrity in the event of an accident. The tests were performed all the time using the remote emergency stop system introduced in the Emergency Stop Systems section. The vehicle is driven from the Demonstrator area to the factory hall entrance using its AD functionalities (see Fig. 18).

**Figure 19:**
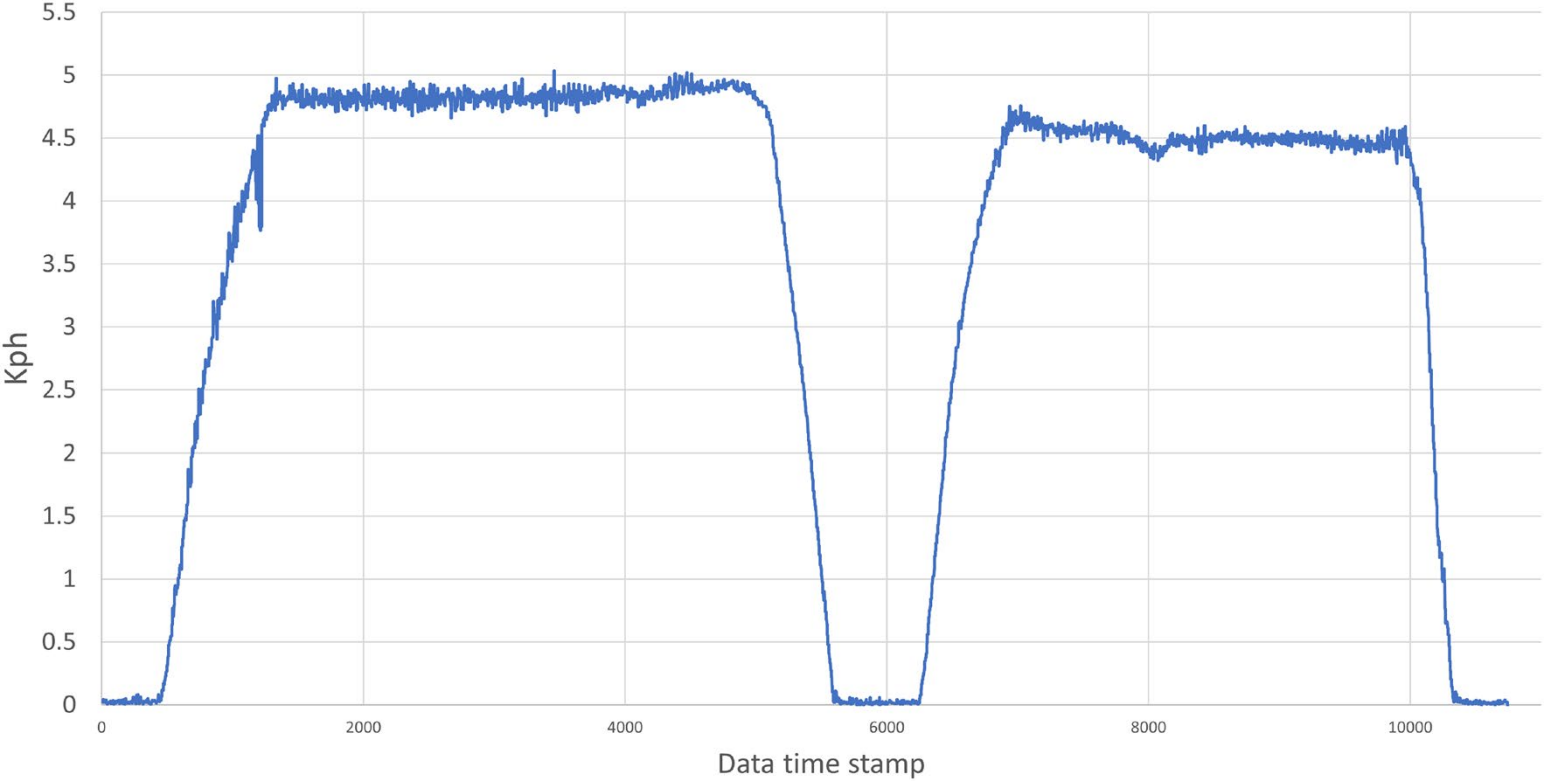
Vehicle speed during the route.

During the AD test, dynamic variables and sensor data were recorded for post-analysis at a frequency of 100 Hz while driving the SDVP inside the factory hall. A total of 10,746 data points were collected from the start to the end of the test. The vehicle’s maximum speed is limited to 5 kph, as shown in Fig. 19. The speed control system effectively maintains the vehicle speed below the speed limit during the test. The average, maximum, and minimum speed of the vehicle during the trip is summarized in Table 1.

As shown in Fig. 19, the vehicle stops when it detects an obstacle, this occurs in the middle of the trip and can be seen in the speed plot. The vehicle sends the stop signal when an obstacle is detected 4 meters in front of it, therefore, the vehicle reaches a maximum braking distance of 1.5 meters when going at a speed of 5 kph during the test. After the obstacle is cleared, the vehicle continues on its way to the entrance of the factory hall.

**Table 1:** Vehicle speed statistics during the final driving test.

Speed [kph]	
Avg.	3.59
Max.	5.03
Min.	0

**Figure 20:**
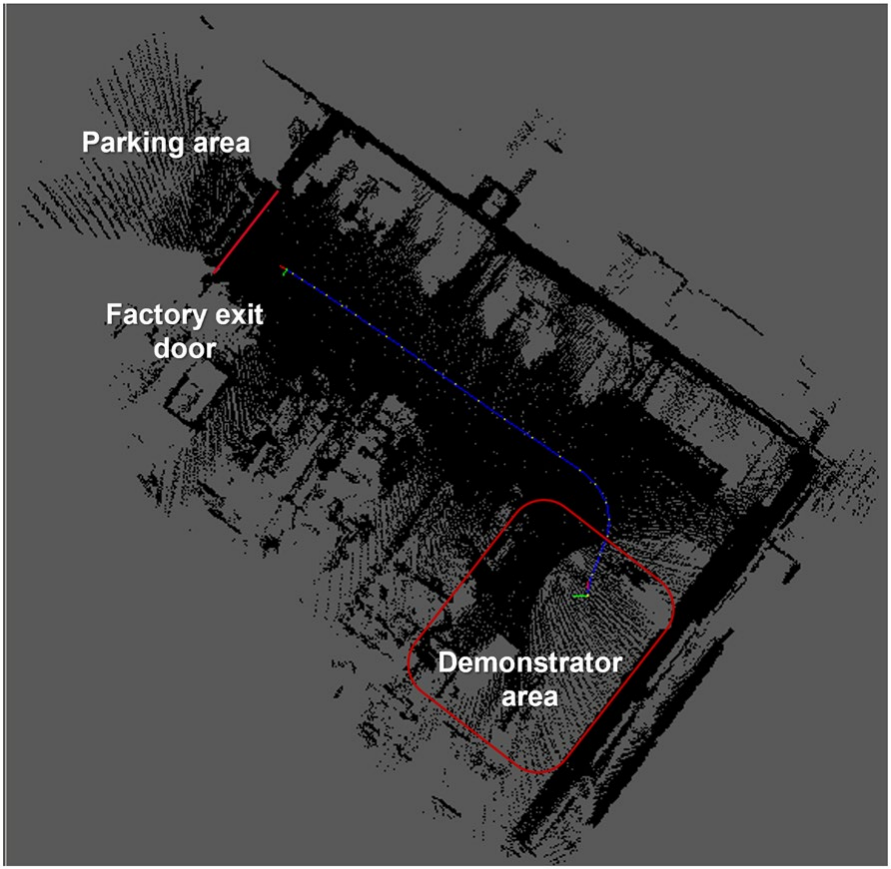
Localization and tracking of the vehicle inside the factory.

On the route, the localization of the vehicle was validated using the generated map through the sensor setup installed in the vehicle and the localization algorithms described in the Vehicle Localization and Mapping section. This allows the vehicle to be localized within the factory and to navigate safely throughout the route (see Fig.  20). UWB technology allows us to improve vehicle localization and tracking within the factory environment by fusing the data from receiver tags and SLAM algorithms.

**Figure 21:**
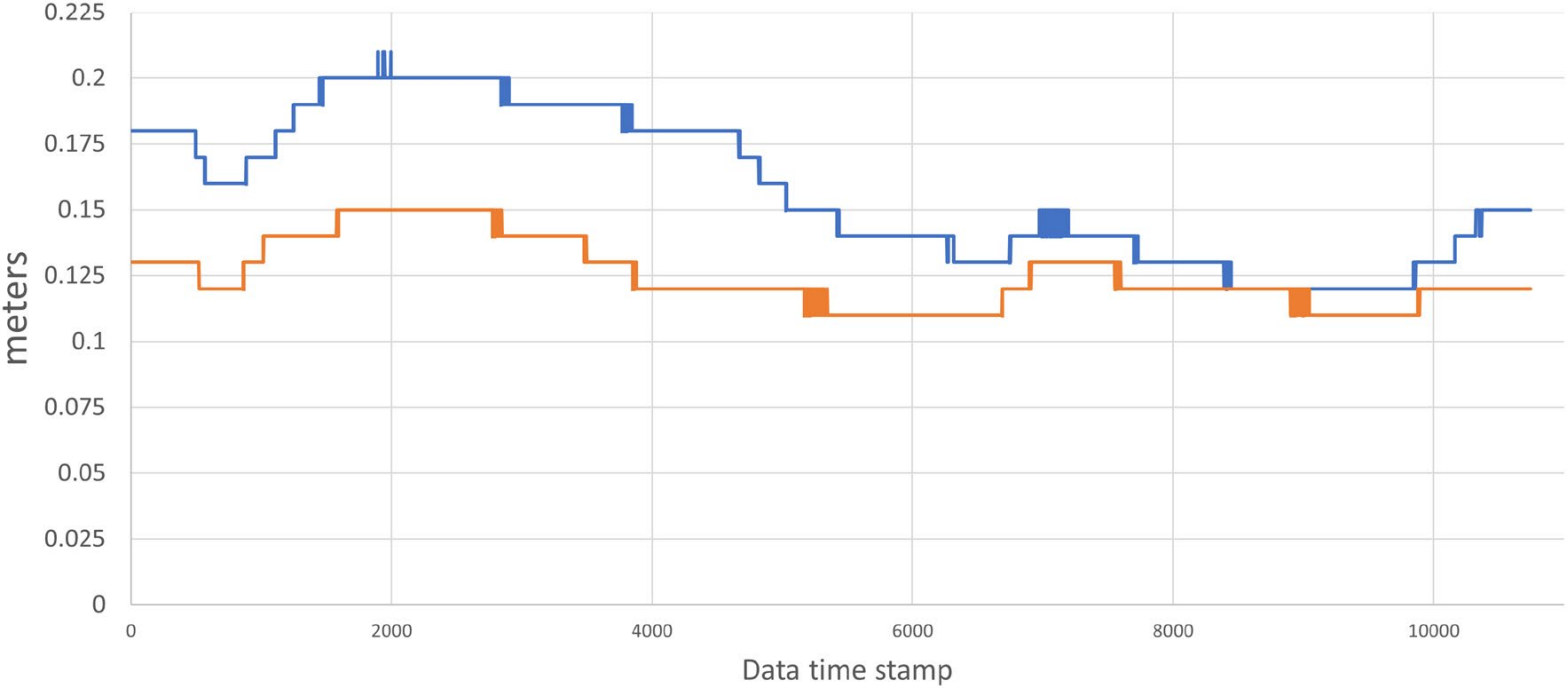
Localization standard deviation in X (Blue) and Y (Orange) coordinates.

The installed systems allow us to calculate the estimated standard deviation error during vehicle localization. This value is given in relation to the X and Y coordinates of the vehicle during navigation (see Fig. 21). During the vehicle trip, a maximum error of 21 cm in X and 15 cm in Y is obtained (see Table 2) due to the effective data fusion between the UWB modules and the accuracy of the odometry during the vehicle trip.

**Table 2:** Localization standard deviation error.

Standard deviation [m]		
	*X*	*Y*
Avg.	0.158	0.125
Max.	0.21	0.15
Min.	0.12	0.11

During the tests, the vehicle receives the start and stop control signal from the CS installed in the Demonstrator area. The communication using MQTT, and the design features of the CS are presented in the Control Station and Communication section. At the end of the test, the vehicle achieves automated driving between the start and end points within the factory. The vehicle constantly monitors its speed and corrects its trajectory if necessary.

## Discussion

The results obtained from the prototype vehicle have successfully validated the conversion of the electric vehicle to a self-driving vehicle platform (SDVP) through the use of installed actuators and processing systems. Safety elements such as the emergency stop system have been integrated to ensure the safety of the car’s operation. Dynamic driving tests were conducted to evaluate the motion behavior and response of these systems while driving at low speeds on the road. The performance of the vehicle was validated through the development of various navigation algorithms and simulation tests using state-of-the-art technology. These results are translated into effective vehicle control at a defined speed of 5 kph and a braking response of 1.5m distance.

Localization within the factory is achieved through the use of SLAM algorithms and UWB technology, resulting in a localization error of less than 21 cm while driving. This level of precision is crucial in ensuring safety within the factory assembly area, or route at the end of the production line. The car’s odometry was also precisely captured while navigating, allowing for detailed visualization of the vehicle’s dynamic movements within the generated map.

Results from the communication tests between the CS and the test vehicle validated the integration of the MQTT protocol in the operational scenario. The communication was secure and stable within the area, allowing for the activation and deactivation of systems in the SDVP.

The test drives conducted with the SDVP demonstrated that automated driving could be achieved within the demo factory environment. The proposed scenario allows for the vehicle to be connected, monitored, and controlled by a remote operator at the end of its production line. Despite the successful results achieved, further work is required to optimize and improve vehicle navigation.

The results obtained from this driving test provide valuable insights into the use of self-driving platforms in assembly systems and end-of-line transport. The successful conversion of the electric vehicle to an SDVP highlights the potential for increased efficiency and reduced operating costs in vehicle manufacturing. Furthermore, the validation of various navigation algorithms and the integration of safety elements and communication protocols serve as critical steps toward the development of fully autonomous vehicles in industrial environments.

While our results primarily focus on the successful conversion of the electric vehicle to a self-driving vehicle platform (SDVP), it is necessary to consider the energy implications. Integrating autonomous driving technologies not only optimizes efficiency but also presents opportunities for energy optimization and management. Our work exemplifies the automation of vehicles leaving the production line from the point of view of logistics, application, and improvements to complement automated post-production processes. However, the development case can cover even more application areas such as energy management systems, and thus explore the possibilities of efficient vehicle charging in the factory environment.

Specifically, in the open literature, there are various approaches and technologies available for optimizing energy usage during the charging process^56^, such as intelligent charging algorithms, peach-shaving techniques, and integration with other renewable energy sources. This broadens the scope of our findings, emphasizing the potential for sustainable manufacturing practices in the field of autonomous industrial transport.

### Findings

In summary, the general findings and learnings from the described experimentation with the SDVP demonstrator are:

- Successful conversion of an electric vehicle to a self-driving vehicle platform (SDVP) is possible through the use of installed actuators and processing systems.
- Safety elements such as the emergency stop system are crucial for ensuring the safety of the vehicle’s operation.
- Dynamic driving tests and simulation tests using state-of-the-art technology can evaluate the performance of the SDVP.
- Precise localization of the vehicle within the factory is possible through the use of SLAM algorithms and UWB technology.
- The generation of a high-quality map is a critical component of any localization and mapping system, and it has a direct impact on the accuracy and reliability of the overall system.
- Communication tests have validated the integration of the MQTT protocol in the operational scenario, allowing for the activation and deactivation of systems in the SDVP.
- Automated driving can be achieved in assembly systems and end-of-line transport, potentially leading to increased efficiency and reduced operating costs in vehicle manufacturing.
- Further work is required to optimize and improve vehicle navigation in SDVPs.
- The successful conversion of the electric vehicle to an SDVP and the validation of various navigation algorithms and safety elements serve as critical steps toward the development of fully autonomous vehicles in industrial environments.

Overall, the demonstrator highlights the potential of SDVPs in industrial settings and the importance of continued research and development in this area to improve the safety, efficiency, and effectiveness of these systems.

## Conclusion and future work

The increasing availability of autonomous driving and driver assistance systems in modern vehicles presents an opportunity to utilize these technologies from the moment the cars are produced. In this study, we present a connected automated vehicle (CAV) demonstrator that exemplifies the integration of these technologies in the end-of-line production transport of electric cars. The CAV demonstrator was developed by designing and integrating various actuators, processing systems, and control subsystems, enabling effective automated driving within the defined operating design domain (ODD) with the help of sensor instrumentation, processing systems, and navigation algorithms. The final demonstrator operates in an environment where the vehicle is continuously connected via WiFi and MQTT protocols for message management, enabling remote monitoring and control of the vehicle within its operation area.

The effective incorporation of autonomous driving systems into assembly processes and end-of-line transportation stands as a significant advancement in vehicle manufacturing. This integration offers the promise of enhancing operational efficiency and cutting down on operating costs. As a result, the showcase of the Autonomous Vehicle Transport Concept within an industrial operating design domain (ODD) and the development of the connected automated vehicle (CAV) demonstrator constitute substantial contributions within the scope of this study.

In summary, while this study showcases the successful integration of autonomous driving and driver assistance systems in the end-of-line production transport of electric cars, it also underlines the potential for increased efficiency, safety, and reduced operating costs in vehicle manufacturing. Nevertheless, ongoing research is essential to enhance safety for higher vehicle speeds, making it a more lucrative business case for manufacturers, especially considering the current high cost of technical equipment. The incorporation of advanced driver assistance systems in production cars implies a future where these technologies become integral to automotive manufacturing processes.

In future work, we plan to further detail the technical and algorithmic operation of car navigation, as well as the difficulties and limitations of autonomous driving in this kind of ODDs. We also aim to modify the car’s sensor configuration to achieve a more standardized architecture as used in production cars, thereby validating the car’s navigation in the industrial ODD with this standardized sensor setup.

## Data Availability

The code alongside the datasets used and generated during the current study are available from the corresponding author on reasonable request.
